# Burden of disease in Thailand: changes in health gap between 1999 and 2004

**DOI:** 10.1186/1471-2458-11-53

**Published:** 2011-01-26

**Authors:** Kanitta Bundhamcharoen, Patarapan Odton, Sirinya Phulkerd, Viroj Tangcharoensathien

**Affiliations:** 1 1International Health Policy Program(IHPP), Ministry of Public Health, Nonthaburi, Thailand

## Abstract

**Background:**

Continuing comprehensive assessment of population health gap is essential for effective health planning. This paper assessed changes in the magnitude and pattern of disease burden in Thailand between 1999 and 2004. It further drew lessons learned from applying the global burden of disease (GBD) methods to the Thai context for other developing country settings.

**Methods:**

Multiple sources of mortality and morbidity data for both years were assessed and used to estimate Disability-Adjusted Life Years (DALYs) loss for 110 specific diseases and conditions relevant to the country's health problems. Causes of death from national vital registration were adjusted for misclassification from a verbal autopsy study.

**Results:**

Between 1999 and 2004, DALYs loss per 1,000 population in 2004 slightly decreased in men but a minor increase in women was observed. HIV/AIDS maintained the highest burden for men in both 1999 and 2004 while in 2004, stroke took over the 1999 first rank of HIV/AIDS in women. Among the top twenty diseases, there was a slight increase of the proportion of non-communicable diseases and two out of three infectious diseases revealed a decrease burden except for lower respiratory tract infections.

**Conclusion:**

The study highlights unique pattern of disease burden in Thailand whereby epidemiological transition have occurred as non-communicable diseases were on the rise but burden from HIV/AIDS resulting from the epidemic in the 1990s remains high and injuries show negligent change. Lessons point that assessing DALY over time critically requires continuing improvement in data sources particularly on cause of death statistics, institutional capacity and long term commitments.

## Background

Disability Adjusted Life Year (DALY) became widely known in the 1993 when the World Bank introduced this term in its report 'Investing in Health', as a summary measure of population health for priority setting [1]. DALY was recommended as an improved measure because it combined fatal and non-fatal health outcomes. It facilitates comparison of burden of diseases (BOD) across different diseases and conditions and is useful in measuring the magnitude and profile of BOD either cross sectional or time trend comparisons, providing evidence for priority settings. It also forms basis for selection of conditions and interventions for cost effectiveness assessment [2-5].

Since the introduction of global estimates [6,7], there had been a number of countries, mostly developed, conducting national level BOD, for example, Australia, New Zealand, USA and Mexico [8-11]. Some assessed BOD on specific diseases, e.g. Korea and Sudan [12,13], or sub-national level, for example, Switzerland and Taiwan [14,15]. Some only estimated number of Year of Life Loss (YLL) such as Burkina Faso due to lack of accurate morbidity data[16]. Nonetheless, a few developing countries demonstrated capacities to sustain BOD assessment though many studies were small scale assessment or part of academic exercises [12,16,17]. BOD estimations are far from complete around the world, especially in developing countries.

Conducting BOD assessment requires tremendous amount of epidemiological data. In developing countries, mortality data are often not existing or incomplete; causes of death are inaccurate; incidence and prevalence of illnesses are not routinely collected and accurately reported. Duration of people living with disabilities in each disease sequel is not possible without a cohort study to monitor disease progression. All these posed limits on BOD assessment in developing countries. Clearly these demands for data require significant institutional capacity to establish and sustain vital registration and key morbidity reports, for which these capacities were not fully developed or supported in many country settings.

Thailand, a middle income country with reasonable data collection system had been successful in producing national level BOD assessment [18-20]. A crucial limitation is a considerable proportion of mis-classification of causes of death (COD) in vital registration. A study on BOD in 1996 adjusted COD from vital registration by modelling and squeeze algorithm [18]. In 1999 the first nation-wide verbal autopsy (VA) study was initiated to verify COD reported by vital registration [21]. Results from this study provided essential information for the 1999 national level BOD assessment [19,20].

While the 1999 BOD study overcame limitation in COD estimates, morbidity data on a small number of diseases were assessed of poor quality [19]. In 2004, based on improved morbidity data of certain diseases, we updated and produced the 2004 national BOD.

This study assesses the national level BOD in Thailand in1999 and 2004 with the application of the 1999 VA to adjust COD in 1999 and 2004 and then compares them in order to contribute to priority setting in health sector investment and program reorientation. Furthermore, this study draws lessons for other developing countries in their efforts to conduct the national BOD assessment.

## Methods

**Figure 1:**
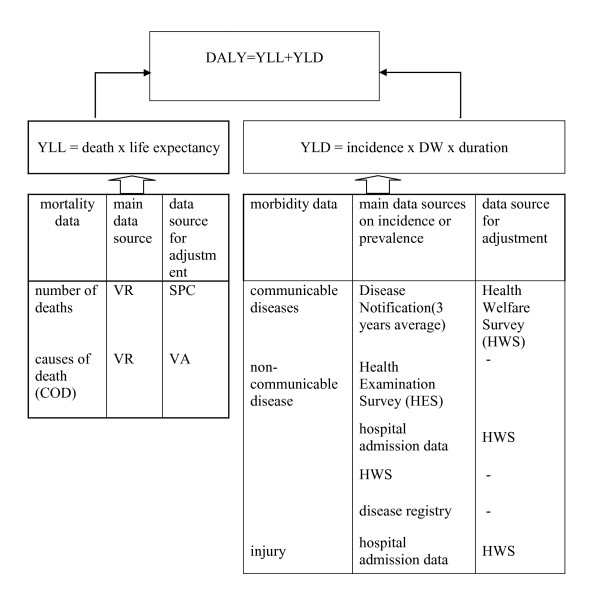
Methodological framework

The study largely employed the methodology recommended by the Global Burden Disease study (GBD) [22] and the Australian burden of disease study [23] detailed elsewhere. DALY is a sum of the years lost due to premature deaths (YLLs) and years lived with disability condition (YLDs). YLLs were calculated with reference to Coale and Demney model life table West Level 26 [24,25]. YLDs are summary of numbers of years in each disabling conditions using disability weight (DW) to represent severity of such conditions. See figure 1

However, there were a number of modifications from the GBD. First, disability weights (DW) were mostly derived from the GBD with a few exceptions where the Dutch weights were applied instead due to the fact that we classified disease sequel according to the Australia's study which then used the Dutch weights. Second, we applied similar discounting rate of 3% but chose not to apply the age weights as in the GBD for the reason that children and older people should not be valued less than adults.

### Selection of causes, age group

GBD classified diseases into three levels of detail: group I (infectious diseases, maternal, peri-natal and nutritional disorders), group II (non-communicable diseases) and group III (injuries). These three groups are further divided into 22 subcategories and classified into more specific conditions at level 3. We modified the lists of disease classification in GBD to be more relevant to the Thai epidemiology situation. Diseases deemed significant to health policy, e.g. dengue and leptospirosis were included in the list. As a result, 110 specific diseases were finally selected. Ages were classified similar to GBD classification (0-14, 15-29, 30-44, 45-59, 60-69, 70-79, 80+).

### Mortality estimation

Mortality data were obtained from national vital registration. By Law, Thailand established civil registry system where all births and deaths were mandatory registered through a nation-wide coverage of civil registration system. Coverage of death registration improved from 75-77% of total deaths in 1985-86 to 95% in 1995-96 according to the mid census Survey of Population Change (SPC) conducted by National Statistical Office [26]. We applied direct method technique for adjusting the completeness of death registration, using trend reported by the SPC [22,26,27].

### COD estimates

Although coverage of death registry was high, COD was assessed of low quality [28]. The 1999 VA study covered 16 representative provinces (out of total 76); interview was conducted to a close relative of the total 43,790 deceased, COD was also verified with medical records in hospitals in which the deceased had a record [21]. Assuming verified COD from VA study is accurate for each COD certified by the official vital registration; correcting factors were generated from the distribution of verified causes in each cause assigned in official mortality records by age group, sex, and region [19]. For missing correction factors in some sub-groups, factors in the wider groups were applied. For example, we divided age into two classifications: 5-year categories (0, 1-4, 5-9, ..., 85+) and GBD larger age category, if there were no correction factors in the small age group, the relevant factors in larger age group were used instead.

### Morbidity estimation

Data required for the YLD estimates consist of incidence, duration of illness, or living with disability, and related disability weights. Extensive reviews of local data sources were performed to derive the best epidemiological estimates. Sources of data included disease notification for infectious diseases in the national epidemiological surveillance system, health measurement survey for particular non-communicable diseases, household health interview survey, hospital admission database, e.g. severe injuries, and small scale survey for those diseases with no other available data sources.

All data sources used in the study are not openly available to the public. The data were obtained by formal permission from respective data authorities. The study was conducted under the supervision of the project steering committee comprising major concerned divisions in the Ministry of Public Health, and experts from a number of Universities in Thailand.

Adjustment to account for coverage of individual data sources was conducted. For example, hospitalization data were extrapolated to compensate with coverage of the data for different types of hospital. Diseases notification data were adjusted for their non-reporting and under coverage in particular from private sector. Validations of the data were undertaken by critical examining the incidence and prevalence against values in other countries and nearby regions. DisMod II [29], a software application provided by WHO, was employed to check internal consistency of parameters estimated. We presented sources of data, estimation approaches and results to disease expert groups in a series of meetings as external review processes.

## Results

### BOD in 2004

Total disease burden in 2004 amounts to 5.7 million DALYs in men and 4.2 million DALYs in women. Fatal burdens is the major component of BOD, it responsible for 69% of total DALY loss in men and 62% in women. Top twenty causes of DALYs loss accounted to 70 and 64 percent in men and women respectively. HIV/AIDS was the leading cause of DALY and YLL in men, while stroke caused the highest DALYs and YLL among women. Traffic accidents and HIV/AIDS were the second leading causes of DALY in men and women respectively. Other conditions in the top five ranking were stroke, alcohol dependence/harmful use, and liver cancer in men; and HIV/AIDS, DM, and depression in women, see Table 1.

**Table 1:** Top twenty causes of DALY loss, 2004, Thailand

Top 20 ranking in men	Death (x1,000)	YLLs (x1,000)	YLDs (x1,000)	Top 20 ranking in women	Death (x1,000)	YLLs (x1,000)	YLDs (x1,000)
1. HIV/AIDS	26.4	634.2	17.7	Stroke	26.1	267.0	48.5
2. Traffic accidents	23.5	548.6	42.7	HIV/AIDS	11.0	279.5	15.1
3. Stroke	23.8	282.6	54.0	Diabetes	14.0	183.7	108.8
4. Alcohol dependence/harmful use	1.0	18.1	315.2	Depression	0.0	0.0	191.5
5. Liver cancer	18.8	277.3	3.1	Ischaemic heart disease	11.5	129.6	10.7
6. Ischaemic heart disease	13.2	168.4	15.6	Osteoarthritis	0.2	1.2	129.9
7. COPD	13.5	124.8	58.6	Traffic accidents	5.1	115.4	10.8
8. Diabetes	8.2	101.6	79.3	Liver cancer	8.7	123.9	1.7
9. Cirrhosis	8.2	140.5	4.3	Deafness	-	-	110.7
10. Depression	-	-	136.9	Anaemia	0.0	0.2	109.3
11. Bronchus & Lung cancer	8.4	109.6	1.5	COPD	5.5	53.2	55.4
12. Homicide and violence	4.1	94.5	13.6	Anxiety disorders	-	-	104.8
13. Deafness	-	-	108.1	Asthma	1.1	15.2	78.7
14. Suicides	4.9	105.7	0.9	Lower respiratory tract infections	6.7	88.8	4.8
15. Lower respiratory tract infections	6.6	99.7	5.3	Dementia	1.7	12.0	70.8
16. Tuberculosis	5.8	77.2	10.4	Cataracts	-	-	81.6
17. Asthma	0.8	11.2	76.1	Cervix uteri cancer	4.5	74.0	1.9
18. Osteoarthritis	0.1	0.4	85.6	Nephritis & nephrosis	5.7	71.7	2.9
19. Anaemia	0.0	0.4	84.3	Breast cancer	3.2	59.5	2.6
20. Drownings	3.2	79.0	0.2	Cirrhosis	3.5	55.4	0.9
All causes	235.3	3948.8	1736.9	All causes	176.4	2592.9	1576.1

### BOD changes between 2004 and 1999

The total numbers of DALYs loss in 2004 were a little higher than those in 1999 both in men and women. Non-communicable diseases contributed to DALYs larger than group I (Communicable, nutritional, childhood and maternal diseases) and group III (injuries). DALYs loss from Group I decreased in 2004, mainly due to reduction in HIV/AIDS deaths; an effect of implementing universal access to ARV since 2002. Burden attributed to non-communicable disease (Group II) increased largely due to demographic change. Burden from injuries showed a negligible increase from 1999.

There was a slight decrease in the DALY loss per 1,000 population (so called DALY rate) among men, from 187.6 in 1999 to 183.8 in 2004. In contrast, DALY rate slightly increased among women, from 129.3 in 1999 to 132 in 2004. See Table 2 and 3. The proportion of burden from premature deaths in 2004 decreased among men, from 75% in 1999 to 69% in 2004, and from 65% to 62% among women, indicating increasing proportion of non fatality conditions for which people lived in disabilities. Burden attributable to HIV/AIDS considerably reduced from 32.3 to 21.1 DALYS per 1,000 population in men but moderately reduced in women (12.2 to 9.3). Proportion of YLL to total DALY due to HIV/AIDS in women slightly increased, but opposite trend among men; this is likely reflecting higher incidences in line with the projection showing second wave of epidemic spread by sexual relation with long-term female partners. Apart from non-fatal diseases, DM, COPD, and Asthma had lower share of burden from premature deaths ranging from 49 to 68 per cent. Drowning and liver cancer had the highest share of burden from premature deaths in men and women respectively.

**Table 2:** Twenty leading causes of DALY (thousands), Thai men, 1999 and 2004

		1999				2004			
		
ranks	Diseases	DALYs	% of total	DALY rates	YLLs/DALYs	DALYs	% of total	DALY rates	YLLs/DALYs
1	HIV/AIDS	960	17.2	32.3	0.98	652	11.5	21.1	0.97
2	Traffic accidents	511	9.2	17.2	0.93	591	10.4	19.1	0.93
3	Stroke	268	4.8	9.0	0.89	337	5.9	10.9	0.84
4	Alcohol dependence/harmful use	131	2.3	4.4	0.18	333	5.9	10.8	0.05
5	Liver cancer	248	4.4	8.3	0.99	280	4.9	9.1	0.99
6	Ischaemic heart disease	164	2.9	5.5	0.94	184	3.2	5.9	0.91
7	COPD	157	2.8	5.3	0.71	183	3.2	5.9	0.68
8	Diabetes	168	3.0	5.7	0.54	181	3.2	5.8	0.56
9	Cirrhosis	118	2.1	4.0	0.97	145	2.5	4.7	0.97
10	Depression	96	1.7	3.2	0.00	137	2.4	4.4	0.00
11	Bronchus & Lung cancer	106	1.9	3.6	0.98	111	2.0	3.6	0.99
12	Homicide and violence	156	2.8	5.3	0.98	108	1.9	3.5	0.87
13	Deafness	94	1.7	3.1	0.00	108	1.9	3.5	0.00
14	Suicides	148	2.7	5.0	1.00	107	1.9	3.4	0.99
15	Lower respiratory tract infections	83	1.5	2.8	0.86	105	1.8	3.4	0.95
16	Tuberculosis	94	1.7	3.1	0.91	88	1.5	2.8	0.88
17	Asthma	49	0.9	1.7	0.24	87	1.5	2.8	0.13
18	Osteoarthritis	94	1.7	3.2	0.00	86	1.5	2.8	0.00
19	Anaemia	88	1.6	2.9	0.01	85	1.5	2.7	0.00
20	Drownings	99	1.8	3.3	1.00	79	1.4	2.6	1.00
									
	Group I	1622	29.1	54.5	0.89	1241	21.8	40.1	0.85
	Group II	2925	52.4	98.3	0.61	3379	59.4	109.2	0.57
	Group III	1034	18.5	34.8	0.93	1066	18.7	34.5	0.90
	All causes	5581	100	187.6	0.75	5685	100	183.8	0.69

Note * DALY rate per 1,000 population

Group I: infectious diseases, maternal, peri-natal and nutritional disorders

Group II: non-communicable diseases

Group III: injuries

**Table 3:** Twenty leading causes of DALY (thousands), Thai women, 1999 and 2004

		1999				2004			
		
ranks	Diseases	DALYs, 1999	% of total	DALY rates	YLLs/DALYs	DALYs, 2004	% of total	DALY rates	YLLs/DALYs
1	Stroke	281	7.1	9.2	0.91	315	7.6	10.0	0.85
2	HIV/AIDS	373	9.4	12.2	0.92	295	7.1	9.3	0.95
3	Diabetes	267	6.8	8.8	0.62	293	7.0	9.3	0.63
4	Depression	145	3.7	4.8	0.00	192	4.6	6.1	0.00
5	Ischaemic heart disease	110	2.8	3.6	0.94	140	3.4	4.4	0.92
6	Osteoarthritis	118	3.0	3.9	0.00	131	3.1	4.1	0.01
7	Traffic accidents	115	2.9	3.8	0.92	126	3.0	4.0	0.91
8	Liver cancer	118	3.0	3.9	0.99	126	3.0	4.0	0.99
9	Deafness	88	2.2	2.9	0.00	111	2.7	3.5	0.00
10	Anaemia	113	2.9	3.7	0.00	109	2.6	3.5	0.00
11	COPD	93	2.4	3.1	0.60	109	2.6	3.4	0.49
12	Anxiety disorders	67	1.7	2.2	0.00	105	2.5	3.3	0.00
13	Asthma	44	1.1	1.4	0.23	94	2.3	3.0	0.16
14	Lower respiratory tract infections	85	2.1	2.8	0.88	94	2.2	3.0	0.95
15	Dementia	70	1.8	2.3	0.07	83	2.0	2.6	0.15
16	Cataracts	96	2.4	3.1	0.00	82	2.0	2.6	0.00
17	Cervix uteri cancer	55	1.4	1.8	0.97	76	1.8	2.4	0.97
18	Nephritis & nephrosis	55	1.4	1.8	0.98	75	1.8	2.4	0.96
19	Breast cancer	44	1.1	1.4	0.97	62	1.5	2.0	0.96
20	Cirrhosis	43	1.1	1.4	0.98	56	1.3	1.8	0.98
									
	
	Group I	1016	25.7	33.3	0.76	854	20.5	27.0	0.76
	Group II	2634	66.7	86.3	0.58	3007	72.1	95.2	0.56
	Group III	297	7.5	9.7	0.93	308	7.4	9.7	0.89
	All causes	3946	100	129.3	0.65	4169	100	132.0	0.62

Note * DALY rate per 1,000 population

Group I: infectious diseases, maternal, peri-natal and nutritional disorders

Group II: non-communicable diseases

Group III: injuries

Among the top twenty leading causes of DALYs, it is apparent that HIV/AIDS burden decreased both in men and women. Only three communicable diseases were listed in the top twenty. For men, only suicides and homicide and violence demonstrated decreased burden compared to 1999. For women, anaemia showed slightly decreasing burden. On the other hand, burden from road traffic injuries slightly increased in both men and women. Burden from alcohol dependence/abuse, cirrhosis, and depression in men were much higher in 2004 than 1999; all the rest in the top ten causes in men had moderate increasing trends. For women, HIV/AIDS revealed a considerable decrease from 9% to 7% but not as high as those found in men. Pattern of diseases in the top ten groups in women were almost the same as it was in 1999, only deafness took over cataract in 2004 where all other diseases remained in the top ten although the ranks were slightly changed.

### South East Asia Regional and Thailand estimates in 2004

The most recent 2004 BOD estimates by the WHO grouped countries by WHO region and income per capita, and by the World Bank geographical regions used in the Disease Control Priorities Project (DCPP) in which Thailand is low and middle income countries in South East Asia region, and lower middle income countries in East Asia and Pacific region [30]. Compared to our estimates, DALY rate in Thailand was far different from that of South East Asia (SEA) region and more close to the estimates for East Asia and Pacific (EAP) in DCPP region, namely 265 and 271 in the SEA region and 195 and 180 in the DCPP region for men and women respectively (data not shown) while it was 184 and 132 DALY per 1000 population in men and women in our estimate. For the WHO's country estimates, Thailand lost 232 and 180 DALY per 1,000 for men and women respectively. These differences are partly due to different method to adjust for incompleteness of vital registration on death. In addition, some of morbidity data were derived from different sources.

Disease burden pattern in Thailand is more similar to upper middle income countries that HIV/AIDS showing larger burden than diarrhoeal diseases and TB. Furthermore, HIV/AIDS was not found among the top ten causes of burden in both SEA and EAP regions where Thailand belongs to. Pattern of disease burden in Thailand inclined towards non-communicable diseases, and ageing demographic compared with the SEA region.

## Discussion

This study provided a comprehensive estimate of national burden of disease in two periods using country best available data. The total DALY loss slightly increased from 9.5 millions in 1999 to 9.9 million in 2004. However, the DALYs rate decreased in men but increased in women. In 2004, two thirds of total DALY loss was attributable to premature deaths. Ratio of YLL to total DALYs in 2004 slightly decreased in both men and women with higher percent change in men, 8% than women, 5%. The leading cause of burden in 2004 was attributed to HIV/AIDS and stroke in men and women respectively. With the exception of HIV/AIDS and traffic injuries, other causes in the top ten lists are non-communicable groups for both men and women. This clearly shows double burden [31] pattern whereby chronic diseases burden had a larger share with increasing trend in the last five years while HIV/AIDS and traffic injuries remained high burden.

Pattern of disease burden in this study reaffirms Hill et al [32] findings of epidemiological transition but interrupted by the 1990s HIV/AIDS epidemic. A projection of HIV/AIDS [33] when no universal ARV was assumed shows HIV/AIDS deaths peaked in 2000 and continued to decline thereafter due to success in prevention. Access to ARV started in 2001 and extended to cover about 50,000 persons in 2004 [34]; current estimate of 190,000 persons were enrolled in 2009. ART further reduced HIV mortality.

Apart from HIV/AIDS, other non-degenerative diseases that showed an increased burden were alcohol dependence/abuse and traffic injuries which warrant immediate policy attentions. Although drug dependence/abuse considerable declined in 2004, alcohol dependence/abuse showed a reverse trend. This was considered as repercussion from a national policy of war on drug, leading to undisclosed of drug use as well as substitution between injection drug, methamphetamine and alcohol [35,36].

COD is one of a major challenge in BOD estimates. Although completeness of death registration is as high as 95%, ill-defined COD in vital registration have been relatively high, about 40% of total deaths in the past years [21,37]. When the 1999 BOD study was undertaken, considerable effort was given to the verbal autopsy study in producing correcting factors for adjustments. While the VA study of 2005 had not been available, we adjusted COD based on the 1999 VA.

Adjusting mortality causes lends itself to the accuracy of VA study. Due to detail classification of disease category in BOD estimates, some causes in the VA study had a very few number of deaths; uncertainty of VA results depends on the size of deaths found in each category. In addition, applying correcting factors from the 1999 VA to 2004 vital registration relies on an assumption that pattern of mis-classification in 2004 remained unchanged from that of 1999.

Other limitations are the extensive demand for mortality and epidemiological data which are at times lacking in developing countries [17,38]; also limited institutional capacities to generate BOD and update on a regular basis. In terms of national health policy uses, limitations included poor understanding of the methodology, low involvement of stakeholders, inability of the methodology to capture key non-economic issues and the costs of carrying out the study [39].

Another setback of estimating BOD trends is the lack of, and inconsistency of data sources between the two periods. We recognized that undertaking normative work such as BOD assessment required substantial financial and time resources, institutional capacity and long term commitments. This limitation in particular in developing countries challenges if DALY, a summary measurement of health gap, is a feasible tool for policy decisions and prioritization [3].

While the GBD is useful in providing burden estimates at regional, groups of countries by development and income level, and sometimes down to national level; our results have shown fairly different figures due to different approaches in the estimations and data sources. Regional estimates can also differ from countries in the region. Pattern of BOD in Thailand differed from the SEA Regional estimate but was more close to countries in the EAP region. We recognise the importance for countries to build their own capacity on BOD estimates and national policy utilities.

In 2004 estimate, lack of information is still unresolved problems. The estimation of YLD essentially demands extensive data on proportion of disease sequel, hardly available in developing countries where limited epidemiological studies have been conducted. Even Thailand has fairly advance infrastructure of both data and health service system, there is only a few large scale cohort studies. Therefore, we reasonably rely on information of nature of disease and sequel from developed countries. This is not so difficult for diseases prevalent in developed countries. For sequel of diseases rarely found in developed countries; panel of experts is applied in our estimates.

Although there are improvements or changes in the data available for the 2004 assessment, the observed changes in BOD profile in 1999 and 2004 are due to actual change in epidemiology of a certain number of diseases of which time series data exist. For example, traffic accidents, cancer, and infectious diseases. Also substantial changes in epidemiology on HIV/AIDS mortality existed as mortality and also years of life loss due to opportunistic infections had significantly reduced in 2004 both men and women, after the introduction of universal ART in 2001. Yet YLD estimates for some diseases suffer from inconsistency of the data sources used in the two assessments.

As generating BOD estimates requires considerable resources, it is therefore essential that the results have an impact on health policy. In Thailand, evidence on BOD was disseminated to relevant health policy makers, particularly, the Thai Health Promotion Foundation, and the MOPH. In the national five year health plan and the Thai Health Promotion Foundation's master plan, BOD was referred to and applied as program priority setting.

A number of milestones in policy development in the Thai Health Promotion Foundation were observed[40]. In addition, there were nine national alcohol policies during 2003-2007. In 2007, a national plan to address primary and secondary prevention of chronic NCD called "Thailand Healthy Style Strategic Plan"[41] was formulated as a result of increased awareness among decision-makers.

### Lesson learned

Based on 1999 and 2004 experiences, this study draws a number of lessons for other developing countries facing similar difficulties. Table 4 summarised challenges and achievements in two groups: methodological issues and institutional capacity to generate evidence and translate evidence into policy decisions.

**Table 4:** Summary lessons from conducting 1999 and 2004 BOD, Thailand

	Challenges	Achievements
**1. Methodological issues**		

1.1 Inadequacy of data	Incompleteness of mortality data from routine vital registration	Direct technique estimation of completeness were applied by using time series from the Survey of Population Change
	
	Poor quality of COD in vital registration	Verbal autopsy and medical record investigation were conducted to verify COD from vital registration
	
	Unavailability of a number of morbidity and disability data	Use of regional estimates from literature review and GBD estimates Information from developed country settings were applied in some instances Consensus meeting with disease experts
	
	Inconsistency of data sources over time	Most datasets maintain consistency

1.2 Adaptation on methods	Classification of disease was not particular fit with tropical diseases	Leptospirosis was added but there was no disability weight values in the GBD
	
	Unavailability of DW	Estimation was derived from the close matched conditions

**2. Institutional capacity**		

2.1 Generating BOD evidence	Fragmentation of mortality and morbidity data sources, time consuming to collate datasets	The capacity of the Thai Working Group on BOD hosted by IHPP was gradually institutionalized Oversight committee provides continued and invaluable supports Comprehensive assessment of demographic, epidemiologic, and health services data on mortality and morbidity Trust based networking with data owners, data users, and disease experts Results have been further utilized in cost-effectiveness assessment National financial commitments and support: the 1999 and 2004 BOD assessments were local research grants. Technical supports from WHO and international experts are invaluable.

2.2 Translation BOD into policy decision		BOD were referenced during national policy formulation process and policy documents, e.g. the Thai Health Promotion Foundation master plan and its 2006 annual report Results were applied in prioritizing health investment fostering resources in primary preventions of chronic NCD, in particular tobacco and alcohol Awareness and increased investments in road safety and HIV preventions.

Lessons told us that technical capacity is not too far to reach; there is always a technical solution to a problem. The difficulty is the institutionalization of capacity to generate evidence and translate evidence to policy and prioritization. Local initiation, local funding, long term commitment and ownership by all stakeholders are key determinants of institutionalization of capacities for BOD assessment.

## Conclusion

The study highlights unique pattern of disease burden in Thailand where non-communicable diseases is increasingly the major disease burden while burden from HIV/AIDS is still high due to mortality and significant years of life loss among prime adults. Negligent change on burden from injuries was observed. Regular assessment of DALY requires continuing improvement in data sources particularly on cause of death statistics, and strengthening of institutional capacity to maintain this important normative work.

## Competing interests

The authors declare that they have no competing interests.

## Authors' contributions

KB carried out the design of conceptual approach, data analysis and drafted the initial manuscript. PO carried out the analysis and data compilation. SP coordinated the study and assisted in the analysis. All authors jointly contributed to the revision of the manuscript and discussion of the results. VT oversaw the study, guided the manuscript drafting and finalized the manuscript. All authors approved the final version of the manuscript.

## Pre-publication history

The pre-publication history for this paper can be accessed here:

http://www.biomedcentral.com/1471-2458/11/53/prepub
